# Print copies, ads and perfusion education

**DOI:** 10.1051/ject/2025005

**Published:** 2025-03-07

**Authors:** Raymond K Wong

As the saying goes, “How time flies when you are having fun!”. As I write this editorial, I am starting my 7th year as the Editor-In-Chief and 3rd year with our partner and publisher, EDP Sciences. When I started, I had multiple objectives, and I am pleased to say that many have been accomplished even though several likely took longer than I had intended. One of my primary goals was to establish a modernized website and tools for an on-line only publication. Our at-that-time new publishers helped us tremendously in setting up a new website with all the essential data-related tools expected of journals nowadays. Interestingly, we have always had individual members of our sponsoring society, AmSECT who yearned for the old days when they were able to feel actual, physical paper while reading our journal content. These members expressed a desire to reacquire that experience even if they had to pay an extra charge for it. Well, AmSECT’s Board of Directors (BOD) have heard these yearnings and have responded! Print runs of 250 copies of each issue has just recently been approved! Members will have the option to order print copies of the journal when they renew their memberships for next year at the AmSECT website. Non-members, and non-perfusionists from anywhere in the world can join AmSECT as full or associate members and order print copies of the journal as well. Furthermore, as part of AmSECT’s mission to support both research and education, the BOD has also decided to provide each US-based perfusion program with two hardcopies of each issue published. Thank you AmSECT!

Support for our journal from industry has also strengthened in the last year with a 65% increase in advertisement sales! Such ad sales are important as they are mutually beneficial; companies expand their messaging while the journal has support in covering costs and is able to limit increases to Article Processing Costs (APCs), thus benefiting authors too. Thank you, members of all the companies serving the perfusion community! They will each be provided with free print copies as well (with the ability to order more copies, if desired), so that they can see how nicely their ads are rendered in the print versions of our journal.

Finally, I would like to highlight a pair of letters-to-the-editor in this issue from Mr. Blaine Johnson, MBA, CCP, University of Chicago Medicine. In these letters Blaine presents very good arguments supporting a Centralized Application Service (CAS) [1] and a National Matching Service (NMS) [2], both for United States-based perfusion education programs. As a US perfusion training program director myself, I find these proposals very intriguing indeed. It leaves me pondering how the implementation of these ideas would affect my processes and workflows in recruiting and admitting new students. Having discussed many of the difficulties faced by applicants with my recently admitted students, I can corroborate that Blaine’s assertions are largely valid. It will not be easy establishing a CAS and an NMS for perfusion education in the US, but as Blaine describes, many other professional training programs around the world have been able to establish such services. These two proposals will certainly be food for thought for us program directors and others involved in perfusion education in the US. Blaine is certainly to be applauded for initiating such considerations.
